# Direct percutaneous treatment of iatrogenic superior gluteal artery injury using angioseal closure device: a novel technique

**DOI:** 10.1186/s42155-025-00574-7

**Published:** 2025-07-08

**Authors:** Kheng Song Leow, Arash Jaberi, Robert Beecroft

**Affiliations:** 1 Department of Medical Imaging, Temerty Faculty of Medicine, University of Toronto, Toronto, ON Canada; 2 Department of Vascular and Interventional Radiology, Joint Department of Medical Imaging, University Health Network, Toronto, ON Canada

**Keywords:** Angioseal, Closure, Embolization, Iatrogenic, Superior Gluteal Artery

## Abstract

**Background:**

Iatrogenic superior gluteal artery injury (SGA) following bone marrow biopsy is rare but potentially life-threatening. Due to the deep intrapelvic location of the vessel, conventional management with manual compression or surgical repair is challenging. Traditional management via endovascular coil embolization requires arterial access and vessel sacrifice.

Case presentation

We present a case of SGA injury resulting from a bone marrow biopsy in a patient with suspected T-cell lymphoma. The injury was successfully managed using a 6 french Angioseal closure device applied directly through the biopsy puncture site in the gluteal region, with the patient maintained in the lateral decubitus position. The approach achieved immediate hemostasis while preserving arterial patency.

**Conclusion:**

This represents the first reported use of an Angioseal device for direct percutaneous treatment of iatrogenic SGA injury. This technique offers an effective hemostasis and vessel preservation, expanding the interventional radiology’s armamentarium.

**Graphical Abstract:**

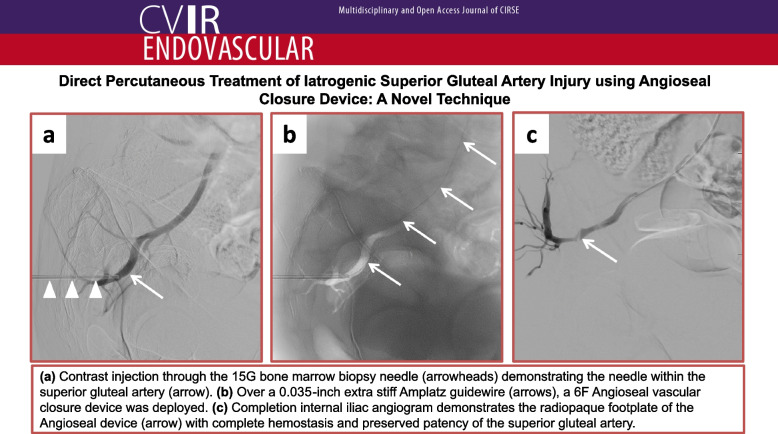

## Background

Bone marrow sampling is a common procedure for diagnosing, staging, and monitoring hematologic diseases including lymphoma, leukemia, and myeloproliferative disorders. Superior gluteal artery (SGA) injury is a rare but potentially life-threatening complication [1]. The SGA emerges from the posterior division of the internal iliac artery, traverses the greater sciatic foramen, and supplies the gluteal musculature. Management challenges include the vessel's deep intrapelvic location, making manual compression ineffective and surgical repair complex [1]. Conventional endovascular coil embolization sacrifices the vessel, risking buttock claudication, while stent grafts may thrombose during sitting [2]. Arterial access is further complicated when patients remain in lateral decubitus position with the biopsy needle in situ. The Angioseal vascular closure device (Terumo Medical Canada) sandwiches arterial puncture sites between an intravascular anchor and extravascular collagen plug, achieving hemostasis while preserving vessel patency [3]. Previous report has described Angioseal use for intentional SGA puncture closure in endoleak embolization post-endovascular aneurysm repair [4]. However, to our knowledge, its application for managing iatrogenic SGA injury has not been described. We present the first reported case of direct percutaneous management of iatrogenic SGA injury using Angioseal device, offering a minimally invasive solution, vessel-preserving solution for this uncommon but serious complication.

## Case presentation

A 34-year-old male presented with a 3-week history of neck pain accompanied by constitutional symptoms including loss of appetite, lethargy, and night sweats. The patient also reported a 1-week history of bilateral upper limb weakness and numbness, more pronounced on the right side, without urinary or fecal incontinence.

Computed tomography (CT) and magnetic resonance imaging (MRI) of the neck revealed an enhancing cervical paravertebral soft tissue mass with extension into the exit foramina and an epidural component causing moderate spinal canal stenosis. Additional findings included splenomegaly with multiple intrasplenic masses, cervical-mediastinal-abdominal lymphadenopathy, and osseous involvement. This constellation of imaging findings was highly suspicious for lymphoproliferative disease.

Due to the intimate relationship of the cervical paravertebral mass with nerve roots and the vertebral artery, the largest cervical lymph node was selected for biopsy. Preliminary histopathology results identified T-cell lymphoma. To guide treatment planning, bone marrow aspiration and biopsy from the right iliac bone was recommended and performed by a member of the hematology team using anatomical landmark without imaging guidance, with the patient positioned in the supine position.

During the procedure, a 15G biopsy needle was inserted too inferiorly, traversing the greater sciatic foramen and puncturing the superior gluteal artery (SGA). The vascular injury was confirmed when pulsatile blood appeared upon partial withdrawal of the inner stylet. The stylet was immediately reinserted, and the biopsy needle was secured at the gluteal region to tamponade the puncture site, with the patient maintained in lateral decubitus position. Blood products were placed on standby, and interventional radiology (IR) was consulted.

**Fig. 1:**
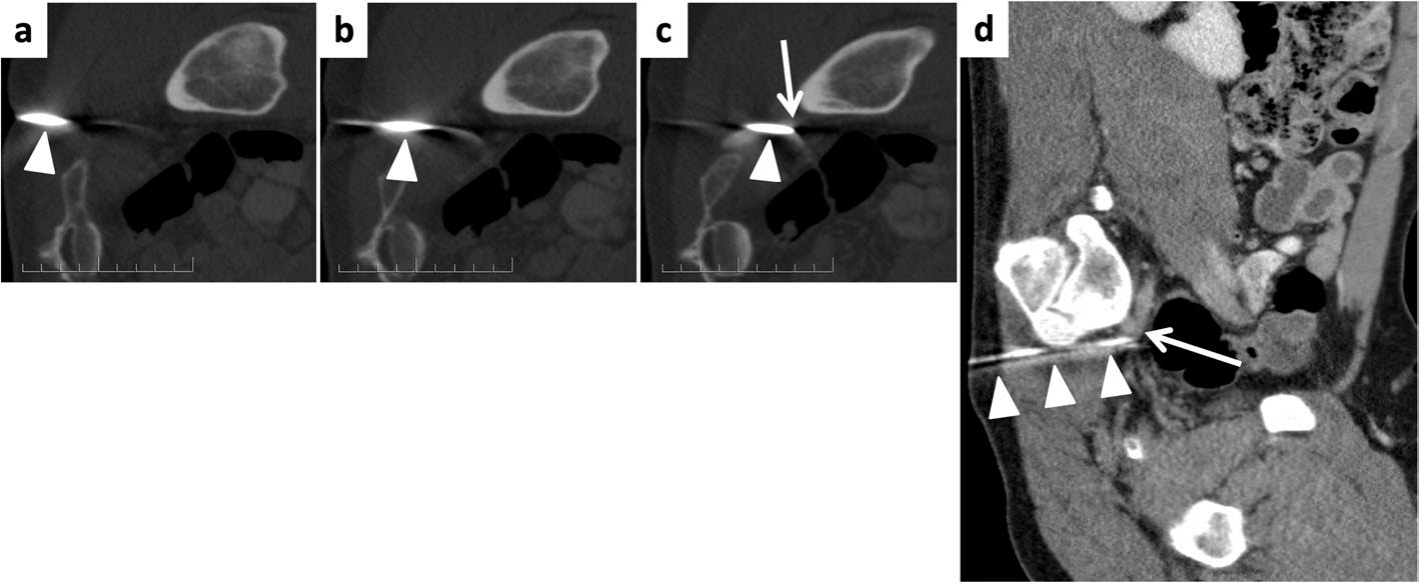
Serial axial (**a**-**c**) coronal (**d**) computed tomography angiography (CTA) with the patient in lateral decubitus position demonstrating the bone marrow biopsy needle (arrowheads) traversing the greater sciatic foramen with its tip within the superior gluteal artery (arrow). Note the absence of surrounding hematoma due to the tamponade effect of the needle

Computed tomography angiography (CTA) confirmed right SGA injury (Fig. 1). The absence of surrounding hematoma was attributed to the tamponade effect of the in situ needle. The patient was transferred to the IR suite, where, after considering various treatment options (endovascular coil embolization, stent-graft placement, and percutaneous gelatin-sponge torpedo), we elected to treat the arterial injury using a 6 F Angioseal closure device.

**Fig. 2:**
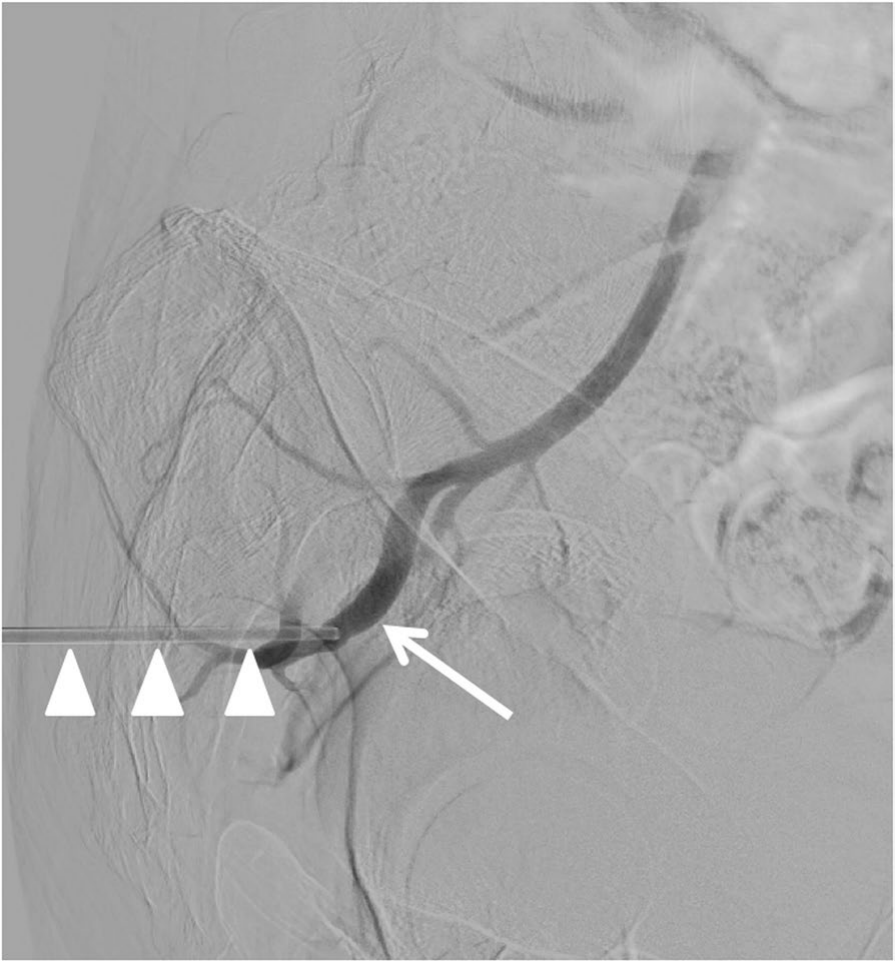
Digital subtraction angiography with the patient in lateral decubitus position. Contrast injection through the outer cannula of the biopsy needle (arrowheads) confirms intraluminal position with opacification of the superior gluteal artery (arrow) and retrograde reflux of contrast into the internal iliac artery

**Fig. 3:**
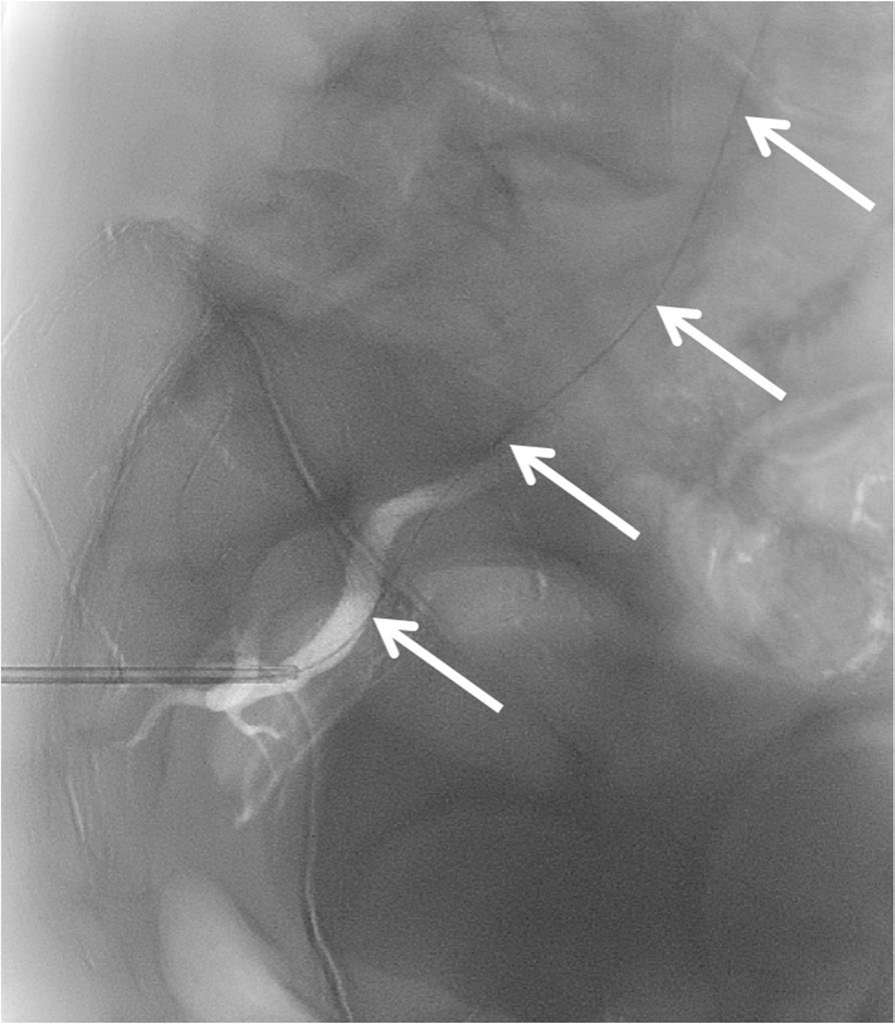
Fluoroscopic roadmap image showing placement of a 0.035-inch extra-stiff Amplatz guidewire (Cook Medical, Bloomington, IN, USA) through the outer cannula of the biopsy needle. The guidewire (arrows) extends cranially through the superior gluteal artery and internal iliac artery into common iliac artery and abdominal aorta, providing enhanced stability during subsequent Angioseal delivery

With the patient maintained in left lateral decubitus position, the inner stylet of the biopsy needle was removed, and contrast injection through the outer cannula confirmed intraluminal positioning within the SGA (Fig. 2). To achieve enhanced access stability due to the underlying gluteal musculature, we substituted the standard 0.035-inch guidewire from the Angioseal kit with a 0.035-inch extra-stiff Amplatz wire (Cook Medical, Bloomington, IN, USA), and advanced the guidewire retrogradely up the SGA and internal iliac artery into the common iliac artery and abdominal aorta (Fig. 3). Over this wire, the 6 F Angioseal device was inserted, and the footplate was deployed followed by the collagen plug according to standard technique. Subsequently, manual compression was applied for five additional minutes, followed by application of a pressure dressing.

**Fig. 4:**
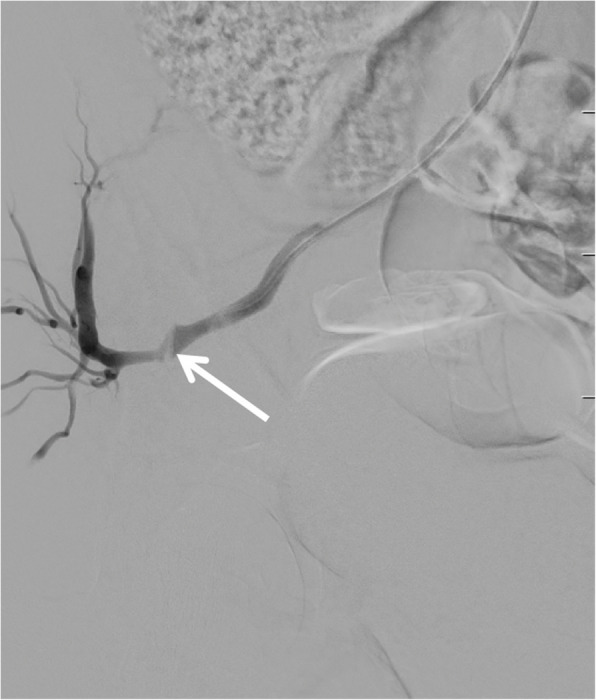
Post-procedural right internal iliac angiogram obtained via contralateral access (left common femoral artery) confirming successful Angioseal deployment with complete hemostasis and no contrast extravasation. Note the linear filling defect (arrow) within the superior gluteal artery representing the footplate of the Angioseal device. Patent superior gluteal artery with preserved distal flow demonstrates successful vessel-sparing intervention

The patient remained hemodynamically stable throughout the procedure. To confirm successful hemostasis, prophylactic angiography of the right internal iliac artery was performed via contralateral (left common femoral artery) access after repositioning the patient from lateral decubitus to supine position. The angiogram demonstrated a linear filling defect within the SGA corresponding to the Angioseal footplate and complete cessation of hemorrhage (Fig. 4). The right SGA remained patent. Lastly, hemostasis of the contralateral femoral access was also achieved with 6 F Angioseal. Patient remained well after 5 days post-procedure. No episode of re-bleeding nor pseudoaneurysm from the contralateral femoral access.

## Discussion

The management of iatrogenic superior gluteal artery (SGA) injury presents distinctive clinical challenges stemming from the vessel's deep anatomical position. This location renders manual compression ineffective and surgical repair technically demanding. Our application of the Angioseal vascular closure device offers an innovative solution for this rare but potentially catastrophic complication of bone marrow sampling. Conventional management through transarterial coil embolization, while hemostastically effective, requires vessel sacrifice with associated risk of buttock claudication [2]. Additionally, standard femoral arterial access becomes particularly challenging when maintaining the patient in lateral decubitus position with the biopsy needle in situ to prevent further hemorrhage. The direct percutaneous Angioseal approach confers multiple clinical benefits: immediate hemostasis via collagen plug tamponade, preservation of arterial patency, treatment with minimal patient movement (reducing risks of needle displacement and exacerbated bleeding), elimination of secondary access sites (reducing their potential complications e.g. pseudoaneurysms) and lastly collagen component of the Angioseal device undergoes complete biodegradation within 90 days (leaving no permanent foreign body within the vessel wall). While vascular closure devices have been employed in other scenarios - including traumatic subclavian artery pseudoaneurysm management [5] and closure of SGA using gelfoam slurry and StarClose for type II endoleaks following endovascular aneurysm repair (EVAR) involving the internal iliac artery [6, 7], our application represents the first documented use for biopsy-related SGA injury. It represents a valuable addition to the interventionalist's armamentarium for addressing this uncommon but potentially life-threatening vascular complication of bone marrow sampling.

## Conclusion

Direct percutaneous embolization of iatrogenic SGA injury using the Angioseal closure device is a novel technique and simple to perform. Though an off-label use, the technique provides a valuable alternative in the IR's armamentarium in cases where conventional endovascular approaches are technically challenging.

## Data Availability

Please see references.
